# Direct-to-consumer advertising of prescription medications: what the Canadian medical trainee needs to know

**Published:** 2019-07-24

**Authors:** Michael D. Elfassy, Sharef Danho, Alexander Adibfar, Joel Lexchin

**Affiliations:** 1 Faculty of Medicine, University of Toronto, Ontario, Canada; 2 Faculty of Health, York University, Ontario, Canada; 3 Emergency Department, University Health Network, Ontario, Canada

You are a medical student in clinic asked to see a 42-year-old man with a past medical history of anxiety. He is presenting with concerns about progressive balding and weight gain. Recently, he came across an internet advertisement suggesting his symptoms may be linked to a low testosterone level. He asks you to provide him with a testosterone prescription. What should medical trainees know about advertising of pharmaceutical products to help patients assess the quality of the information that they have received?

## Current Canadian Regulations

Under the Food and Drug Regulations in Canada, Direct-to-Consumer Advertising of Prescription Medications (DTCA) is generally prohibited.^1^ However, for a variety of reasons, Canadians are increasingly exposed. A review of complaints about DTCA to Health Canada shows that through loopholes in the interpretation of legislation and poor enforcement by Health Canada, pharmaceutical companies have used a variety of different tactics to advertise their products.^2^ One of them is “reminder ads” wherein companies can advertise their brand without naming the purpose of their product, simply requesting consumers to “ask your doctor.” Another strategy termed “help seeking” urges people to visit their doctor to discuss a certain medical condition, without identifying the brand or product. In reality, the pharmaceutical industry combines these maneuvers with blatant violations of regulations, knowing the profit benefit outweighs any possible sanctions or fines. A Canadian example is Diane-35, a drug approved for treatment of severe acne, but where the company used marketing techniques that implied it could be used for off-label contraception and suffered few repercussions for doing so.

In an increasingly globalized world, borders are in many ways beginning to fade. This holds especially true for DTCA, through which broadcast and online use could expose consumers to advertisements that are not bound by Canada’s regulation. Many Canadians already watch US television broadcasts, which contain many poorly-regulated pharmaceutical advertisements.^3^ With digital DTCA on the rise and regulatory responses several steps behind, online platforms are a popular medium for industry investments.^4^ Expenditure on internet-based DTCA is growing, whereas all other categories have been experiencing steady expenditure declines, suggesting a strategic shift in keeping with the evolution of digital health.^5^ More than ever, Canadians are vulnerable to DTCA, and our physicians must be trained to know how to respond when patients make requests based on ads that they have seen.

## Evidence regarding DTCA

While it has been suggested that DTCA may have benefits such as identifying and encouraging treatment for diseases, improving medication compliance, and promoting health education, the bulk of prevailing evidence indicates that these effects are speculative and do not justify harms to public health.^6^ DTCA misleads consumers by obscuring side effects and harms, concealing details about products that may put them in a negative light, and instead presenting information to cast products or health conditions of interest in a positive way.^7^ Patients who encounter these biased advertisements are likely to present to their physicians with questions and requests, and physicians in turn are more likely to order tests or prescribe medications consistent with the advertising.^8^ This can lead to overprescribing, disease mongering, unnecessary medication use, and suboptimal treatment, all of which could have dangerous consequences.^9^

## What is taught in medical school about DTCA

To our knowledge, no Canadian medical school dedicates a formalized curriculum component specific to DTCA. However, Canadian medical schools do have policies towards conflicts of interest; these govern direct interactions between students and pharmaceutical companies as well as indirect interactions between students and lecturers who have a relationship with pharmaceutical companies.^10^ These policies, however, range widely in comprehensiveness across the medical schools – within Canada, different medical schools afford varying levels of protection to students about conflicts of interest that may impact the education that they are receiving.

Taken together, this lack of a strong, uniform focus on policies about conflicts of interest suggests that any teaching regarding DTCA is currently insufficient and that the content is variable among, and likely even within, schools. This is a cause for concern because medical students will likely encounter DTCA early in medical school while interacting with patients. For example, students are frequently reminded that their future patients will likely have “Googled” their symptoms prior to an appointment. Up to 70% of Canadians search their medical symptoms online prior to seeing a doctor.^11,12^ If this is the case, patients will have undoubtedly been subjected to DTCA, as eDTCA is one of the fastest growing forms of DTCA. Data tracking is a growing phenomenon and is re- shaping the advertising field entirely. As such, internet DTCA is more customized than ever, with advertisements tailored to patients’ specific internet browsing history – one click at a time.^5^

## Revisiting our introductory case

Does the case presented above in the introduction warrant testosterone investigation? A 2015 Canadian clinical practice guideline on testosterone deficiency and management does not provide a clear answer.^13^ When clinical guidelines are unclear or potentially ambiguous, physicians can be swayed in favour of marketed/advertised products, especially in the face of interest from a patient. This 42-year-old patient’s symptoms of weight gain and balding could be explained by a host of medical, environmental, and genetic factors. In the case of testosterone advertising in the US between 2000 and 2011, DTCA was associated with an increase of more than 250% in testosterone prescriptions for males.^14^ Testosterone prescriptions were often initiated for non-specific symptoms and even when patients did not meet the classical diagnostic criteria for hypogonadism. Additionally, physicians often deviated from established clinical guidelines by prescribing testosterone without prior serum testing.^15^ Like most other drugs, testosterone is not an inert substance and has been associated with adverse outcomes including myocardial infarction and stroke.^16^ We should therefore always remain vigilant about our prescribing practices, an approach medical students need to learn.

## What medical trainees should know

In the situation where a patient asks about an advertised pharmaceutical product, the medical trainee should remember a useful mnemonic, “DTCA” to structure their approach.

### D – Deliberate

Take a step back and consider how DTCA can influence your own prescribing practices. Be wary that interactions with pharmaceutical companies have been consistently shown to alter the prescribing patterns of physicians, despite the majority denying the capacity to be influenced.^17^

### T – Tune in

Use a patient-centred stance to actively listen to the patient and explore the underpinnings of their request. A strong patient-physician relationship is critical in promoting a candid environment where patients will feel comfortable in discussing and learning about the contents of DTCA.

### C – Counsel

Counsel the patient using an objective approach, taking into consideration the potential benefits, harms, and side effects. Explain that healthcare providers have a fiduciary duty to patients alone, and that pharmaceutical companies do not.

### A – Advise

Offer your perspective and suggest alternative options to present the patient with the necessary information to make an informed decision. After all, the majority of patients want their physicians to help them in understanding the contents of DTCA.^18^

When a patient requests a medication or test that is not in their best interest, it is most advantageous to use strategies that reinforce and validate your patient, while also negotiating the most suitable option. First is the “substitution strategy,” which reframes the diagnosis or offers an alternative treatment. If this fails, the “contingency strategy” can offer a trial of the evidence-based treatment strategy with an agreed-upon definition of success, while agreeing to switch to the patient’s preference after a period of time if the evidence-based treatment is unsuccessful.^19^

## Call to educators

Integration of an approach to a patient with questions about diagnosis and treatment generated by DTCA into all levels of medical training is increasingly important. The same principles hold true for medical devices, genetic testing, and other health products that are directly advertised to patients.
